# Call to Caution with the Use of Atypical Antipsychotics for Treatment of Depression in Older Adults

**DOI:** 10.3390/geriatrics1040033

**Published:** 2016-12-16

**Authors:** Katherine Amodeo, Ruth B. Schneider, Irene Hegeman Richard

**Affiliations:** Department of Neurology, University of Rochester, Rochester, NY 14642, USA; Ruth_schneider@urmc.rochester.edu (R.B.S.); Irene_richard@urmc.rochester.edu (I.H.R.)

**Keywords:** antipsychotic, SNRIs, SSRIs, depression, IPD, DIP, neuroleptic

## Abstract

Atypical antipsychotics are increasingly being used to manage depression in older adults where these symptoms can often be refractory to first-line treatment with selective serotonin reuptake inhibitors (SSRIs) or serotonin norepinephrine reuptake inhibitors (SNRIs). Unfortunately, atypical antipsychotics can be associated with the development of extrapyramidal symptoms (EPS), with drug-induced parkinsonism (DIP) being the most common movement disorder induced by this class of medication. The management of treatment-resistant depression in older adults is of particular concern as depression is a common feature of idiopathic Parkinson’s disease (IPD) and can manifest prior to the development of motor symptoms. Herein, we discuss the use of atypical antipsychotics for the management of depression in older adults including the risk of DIP and propose that antipsychotics may potentially unmask IPD.

## 1. Introduction

Depression is common in the elderly [1]. While the symptoms of depression can be mitigated by treatment, many older adults do not respond to first-line therapy with selective serotonin reuptake inhibitors (SSRIs) or serotonin norepinephrine reuptake inhibitors (SNRIs) [2,3]. Providers are increasingly turning to second-line treatments, including the use of atypical antipsychotics [4,5,6,7]; however, little evidence supports the use of these agents in older adults.

Compared with typical antipsychotics, atypical antipsychotics are less commonly associated with the development of extrapyramidal symptoms EPS. However, the development of drug-induced parkinsonism (DIP), recognized in the Diagnostic and Statistical Manual, 5th edition and International Statistical Classification of Diseases, 10th revision (DSM5/ICD10) by the term “neuroleptic induced parkinsonism”, remains a concern with the use of these medications [8,9]. In addition to the side effect of reversible DIP in the general population of older adults with depression, it is possible that these medications may be associated with long-lasting or permanent symptoms (i.e., tardive parkinsonism).

We put forth the idea that, in susceptible individuals, treatment with atypical antipsychotics may actually “unmask” subclinical or “premotor” idiopathic Parkinson’s disease IPD in patients who have not yet manifested the typical motor features (e.g., tremor, bradykinesia) of the neurological condition [10,11]. It has long been known that the neurodegenerative process begins years prior to the onset of motor features. More recent research has supported the notion that neurodegeneration may present with “premotor” features including anosmia, Rapid eye movement (REM) sleep behavior disorder, and constipation as well as anxiety and depression [12,13,14].

While recognizing that many older adults may fail to respond to first-line treatments for depression, the purpose of this article is to call caution to the use of atypical antipsychotics in this population.

## 2. Depression and the Use of Antipsychotics in Older Adults

### 2.1. Treatment-Resistant Depression among Older Adults

Two percent to 5% of adults aged 65 years or older suffer from major depression [15,16]. Twenty-five percent of suicides are accounted for by those 65 years old or older [17]. Depression is a treatable cause of morbidity and mortality. Of the treatment options, SSRIs and SNRIs are the preferred therapies in this population because of their relatively favorable side effect profiles [18,19]. Unfortunately, depression among older adults is often difficult to manage and can be refractory to these first-line therapies. As many as one-third have treatment-resistant depression [20], with treatment resistance typically defined as an inadequate response to at least one antidepressant of adequate dose and duration [21]. The exact reason for treatment resistance among this population is unclear, but co-morbid diseases or concurrently prescribed medications which can produce depressive symptoms may be among the many possible explanations for perceived treatment-resistant depression [16]. Elderly individuals with depression are more likely to suffer from co-morbid diseases than younger individuals with depression [22,23,24]. There are high rates of depression associated with stroke (30%–60%), coronary artery disease (8%–44%), cancer (1%–40%), Parkinson’s disease (40%), Alzheimer’s disease (20%–40%), and dementia (17%–31%) [22,25].

### 2.2. Use of the Atypical Antipsychotics in the Management of Depression in Older Adults

Atypical antipsychotics have had an expanding role in the treatment of depressive disorders, with a dramatic increase after the US Food and Drug Administration (FDA) approved some agents for this indication in 2006 [26,27]. It is important to note, however, that of the atypical antipsychotics, only quetiapine and aripiprazole have been approved as adjunctive therapies for depressive disorders [26,27]. Asenapine, clozapine, iloperidone, and olanzapine are approved for depressive disorders when used in combination with fluoxetine [26,27]. To date, risperidone and ziprasidone have not yet been approved for use in depression [26,27].

There is evidence to suggest that adjunctive treatment with atypical antipsychotics, including aripriprazole [28,29,30,31], quetiapine [32,33,34], risperidone [35] and ziprasidone [36], may be effective in the management of depression in the non-elderly population. However, there have been few studies examining this approach in the elderly population. Although controversial in the literature, this may, in part, be attributable to the clinically observed increased risk of mortality among older adults treated with antipsychotics [37,38]. While atypical antipsychotics are associated with a lower risk of death than typical antipsychotics, the increased risk of death associated with atypicals remains among patients with certain co-morbidities, namely dementia [37,39]. A recent cohort study reported the relative risk of death with the use of atypical antipsychotics in older adults with dementia versus those not treated with these drugs to be 2.354 [39]. Additionally, atypical antipsychotics, like typicals, carry the increased risk for metabolic syndrome and cardiovascular disease [40]. Therefore, as with all treatment decisions, providers need to consider the possible risks of untreated depression versus the potential benefits with the use of adjunctive therapy. However, there is relatively scarce data upon which to base this decision.

A recent systematic review of the treatment of treatment-resistant depression in the elderly concluded that more high quality, randomized controlled studies are needed [41]. Data from two small, open-label trials [6,7] and from a pooled post hoc analysis suggest that aripiprazole might be effective for the adjunctive treatment of treatment-resistant depression in the elderly [5]. The results of a recent randomized, double-blind, placebo-controlled trial support the conclusion of these preliminary studies [4]. In this trial, patients aged 60 years or older with major depression who failed to achieve remission of depressive symptoms, as defined by a Montgomery Asberg Depression Rating Scale score of 10 or less, following an adequate prospective trial of venlafaxine, were randomized to adjunctive aripiprazole or a placebo. After 12 weeks, 44% of individuals achieved remission of depressive symptoms in the aripiprazole group versus 23% in the placebo group [4]. While the authors report a significant benefit with the use of aripiprazole in older adults with depression, the rate of DIP (as defined by a two-point increase in the total Simpson Angus Scale score) was 17% in the aripiprazole group versus 2% in the placebo group [4]. This finding adds to the existing literature regarding the associated risk of parkinsonism with treatment with antipsychotics.

### 2.3. Drug-Induced Parkinsonism with Use of Atypical Antipsychotics in Older Adults

DIP is the most common movement disorder associated with the use of antipsychotics [42,43,44]. The exact rate, prevalence, and incidence of DIP is unclear because DIP is frequently unrecognized or misdiagnosed as IPD [45]. Advancing age might be associated with an increased risk of DIP [46]. Among Medicare beneficiaries 65 years and older, the use of antipsychotics increases an individual’s risk of developing parkinsonism within one year by 94% (odds ratio 1.94) [47]. While older individuals treated with atypical antipsychotics are 30% less likely to develop parkinsonism than those treated with typical antipsychotics, the risk of parkinsonism is similar between those treated with high doses of atypical antipsychotics and those treated with typical antipsychotics [48]. In a study of quetiapine monotherapy for the treatment of depression in older adults, 14% in the quetiapine group compared with 8% in the placebo group experienced worsening on the Simpson Angus Scale [49].

### 2.4. Depression in Idiopathic Parkinson Disease and Management

The use of atypical antipsychotics in the management of depression is of particular interest in older adults given that depression can be a pre-motor feature of IPD. In “An Essay on the Shaking Palsy”, James Parkinson observed that the motor symptoms of IPD are often complicated by depression [50]. Among patients diagnosed with IPD, approximately 35%–40% have depression [13,22,23,51]. It is now widely recognized that depression is a consequence of the underlying neurodegenerative disease process and often precedes the motor symptoms [52,53]. In support of this, the prevalence of depression is higher in IPD than in other chronic illnesses [53,54]. Using the Montgomery Asberg Depression Rating Scale (MADRS) and the Beck Depression Inventory (BDI) , a recent case-control study demonstrated that the prevalence was higher in IPD patients versus age-, sex-, education-, socioeconomic status–, and cognitive functioning–matched non-IPD controls having chronic medical conditions such as diabetes, hypertension, hypothyroidism, coronary artery disease, and osteoarthritis [54]. In this study, the prevalence of depression was 54.3% in individuals with IPD versus 23.3% in controls (*p* < 0.01) on the MADRS and 49.9% in individuals with IPD versus 23.4% in controls (*p* < 0.05) on the BDI [54].

There is a higher incidence of depression among individuals who are subsequently diagnosed with IPD compared with healthy controls. Shiba et al. found that depression was associated with an increased risk for developing IPD (odds ratio 1.9) compared to age-matched individuals who did not develop IPD [55]. Similarly, a recent study found that 10% of individuals had symptoms of depression up to two years prior to the diagnosis of IPD versus 4% of age- and sex-matched healthy controls [56].

Clinically, depression and IPD can present similarly with shared features, including psychomotor slowing, sleep disturbance, diminished concentration, fatigue and sexual dysfunction, which can make the diagnosis of depression in IPD difficult [55]. The presence of pessimistic thoughts, early morning awakenings, rapid functional deterioration over days to weeks, and suicidal ideations are important indications of depression [57].

The similarities and differences between the quality of depressive symptoms and the severity of depressive syndromes in patients with and without PD has been a topic of research [58]. Some reports suggest that depressed individuals with IPD tend to experience symptoms of dysphoria, irritability, and pessimism at a higher frequency than those with primary depression, while experiencing less guilt or self-blame and delusions [58]. Less than half of individuals with IPD and depressive symptoms have major depression, with most having milder forms of depression [59]. Slaughter et al. report the prevalence of dysthymia, minor depression, and major depression in IPD as 22.5%, 36.6%, and 24.8%, respectively [13]. Similarly, Nuti et al. report the prevalence of major depression and dysthymia as 21.1% and 18.8%, respectively [60]. Anxiety and depression frequently co-occur in IPD [58,60,61]. Of the types of anxiety encountered in IPD, panic disorder, generalized anxiety disorder, and social phobia are most common [60,61].

The literature is clear and consistent, however, with regard to fact that the presence of depression is associated with a poorer quality of life independent of motor deficits in those with IPD [58]. In their study on the prevalence of depression in IPD, Arun et al. found a negative correlation between scores for depression and quality of life (*p* < 0.001) [54]. Diagnosis and treatment of depression are, therefore, crucial.

As in the general population of older adults with depression [2,3], depression in IPD can be difficult to manage. In a large, multi-center, randomized, double-blind, placebo-controlled trial of paroxetine and venlafaxine XR, both medications were effective for the treatment of depression in IPD compared to a placebo. However, one-third of participants in the paroxetine group and one-half of participants in the venlafaxine XR group were considered non-responders [62]. Evidence regarding the use of second-line therapy or augmentation pharmacotherapy in managing refractory depression in IPD is lacking, but the use of antipsychotics should be avoided whenever possible as a worsening of motor symptoms would be expected with the use of a medication that blocks dopamine receptors.

### 2.5. Antipsychotic Use May Unmask Idiopathic Parkinson Disease

It is possible that the development of parkinsonian motor features in some older adults treated for mood disorders with antipsychotic agents may represent an “unmasking” of underlying IPD. It is unclear if exposure to the antipsychotic agent has any impact on the underlying neurodegenerative disease progression, but this is a possibility worthy of consideration.

In animal models, *parkin*-null mice, which have been arguably considered a “susceptible” model for IPD rather than a disease model as they cannot be clinically distinguished from control mice, are more likely to manifest the neurotoxic effects of anti-dopaminergic drugs, suggesting that there is a risk to potentiate parkinsonism or unmask symptoms with exposure to these drugs [63]. Lending support to the concept that antipsychotics may unmask IPD is the observation that parkinsonian symptoms may worsen or progress in individuals who have been exposed to antipsychotics despite cessation of the agent [10,11]. Foubert-Samier et al. performed a 15-year prospective cohort study involving elderly patients and found a 3.2-fold higher risk of Parkinson’s disease among those previously treated with antipsychotics [11].

## 3. Conclusions

Depression is difficult to manage in older adults and there is a high rate of non-response to first-line therapy. More evidence is needed regarding second-line therapy and augmentation pharmacotherapy in this population. Given the considerable risk of parkinsonism with atypical antipsychotic use and the possibility that such agents may unmask subclinical IPD in older adults, the use of antipsychotics for the management of treatment-resistant depression in the elderly should only be undertaken with caution.
